# Deployment of assistive living technology in a nursing home environment: methods and lessons learned

**DOI:** 10.1186/1472-6947-13-42

**Published:** 2013-04-08

**Authors:** Hamdi Aloulou, Mounir Mokhtari, Thibaut Tiberghien, Jit Biswas, Clifton Phua, Jin Hong Kenneth Lin, Philip Yap

**Affiliations:** 1 Image & Pervasive Access Lab, CNRS UMI 2955, Singapore, Singapore; 2 Institut Mines-Télécom, Paris, France; 3 Montpellier Laboratory of Informatics Robotics and Microelectronics, CNRS UMR 5506, Montpellier Cedex 5, France; 4 Institute for Infocomm Research, A*STAR, Singapore, Singapore; 5 Khoo Teck Puat Hospital, Alexandra Health, Singapore, Singapore

**Keywords:** Ambient assistive living, Dementia assistance, Real life deployment, Dynamic and adaptable systems, Context aware services

## Abstract

**Background:**

With an ever-growing ageing population, dementia is fast becoming the chronic disease of the 21^st^ century. Elderly people affected with dementia progressively lose their autonomy as they encounter problems in their Activities of Daily Living (ADLs). Hence, they need supervision and assistance from their family members or professional caregivers, which can often lead to underestimated psychological and financial stress for all parties. The use of Ambient Assistive Living (AAL) technologies aims to empower people with dementia and relieve the burden of their caregivers.

The aim of this paper is to present the approach we have adopted to develop and deploy a system for ambient assistive living in an operating nursing home, and evaluate its performance and usability in real conditions. Based on this approach, we emphasise on the importance of deployments in real world settings as opposed to prototype testing in laboratories.

**Methods:**

We chose to conduct this work in close partnership with end-users (dementia patients) and specialists in dementia care (professional caregivers). Our trial was conducted during a period of 14 months within three rooms in a nursing home in Singapore, and with the participation of eight dementia patients and two caregivers. A technical ambient assistive living solution, consisting of a set of sensors and devices controlled by a software platform, was deployed in the collaborating nursing home. The trial was preceded by a pre-deployment period to organise several observation sessions with dementia patients and focus group discussions with professional caregivers. A process of ground truth and system’s log data gathering was also planned prior to the trial and a system performance evaluation was realised during the deployment period with the help of caregivers. An ethical approval was obtained prior to real life deployment of our solution.

**Results:**

Patients’ observations and discussions allowed us to gather a set of requirements that a system for elders with mild-dementia should fulfil. In fact, our deployment has exposed more concrete requirements and problems that need to be addressed, and which cannot be identified in laboratory testing. Issues that were neither forecasted during the design phase nor during the laboratory testing surfaced during deployment, thus affecting the effectiveness of the proposed solution. Results of the system performance evaluation show the evolution of system precision and uptime over the deployment phases, while data analysis demonstrates the ability to provide early detection of the degradation of patients’ conditions. A qualitative feedback was collected from caregivers and doctors and a set of lessons learned emerged from this deployment experience. (Continued on next page) (Continued from previous page)

**Conclusion:**

Lessons learned from this study were very useful for our research work and can serve as inspiration for developers and providers of assistive living services. They confirmed the importance of real deployment to evaluate assistive solutions especially with the involvement of professional caregivers. They also asserted the need for larger deployments. Larger deployments will allow to conduct surveys on assistive solutions social and health impact, even though they are time and manpower consuming during their first phases.

## Background

The world’s population is ageing rapidly with an estimation of 1 in 5 people over 65 years old by 2030 compared to 1 in 10 today. Due to chronic age-related illnesses, many progressively lose their autonomy and become more dependent on others, finally reaching the stage when they need round-the-clock care from their family members or caregivers. One of the most important chronic disease that affects the ageing population is dementia. It accounts for 4.1% of total disease burden among people aged over 60 years and 40% of people older than 85. The number of people affected by this disease is increasing exponentially with an estimation of 35.6 million people with dementia in 2010, and numbers nearly doubling every 20 years [1].

According to the Global Deterioration Scale (GDS) [2], cognitive and functional abilities are categorized into 7 stages, ranging from no cognitive decline in the first stage to very severe cognitive decline in the seventh stage. Stage 5 denotes the point where it becomes difficult for the patient to live independently and assistance is needed from his/her family and/or caregivers. During stages 3 to 5, patients suffer progressive cognitive decline and experience increasing difficulties in performing activities of daily living (ADLs) [3]. In some instances, patients may understand what they are supposed to be doing but they may not understand the instructions, or forget them midway through a task. They may also fail to recognize objects for what they are (agnosia) or know how to execute learned tasks (apraxia). This means that the caregivers have to be present to support patients during their activities, and slowly, over time, increase the support they provide as the disease evolves. Caregivers need to remain informed on how patients are performing their ADLs, and to provide support as and when appropriate. They need to provide just the right amount of assistance so as not to take over the task, but still allow the patient to retain some level of independence [4]. Over time, as patients need more help, caregivers also experience increasing levels of stress and burden. Caregiving for a dementia patient can be physically and emotionally demanding and has been found to be more stressful than caregiving for older people suffering from other ailments [5].

Ambient Assistive Living (AAL) technologies can be used to assist people with dementia and their caregivers. AAL consists of a set of ubiquitous technologies - e.g. sensors, actuators, interaction devices - embedded in the living space of the patient to monitor and react to his contextual needs by providing computerized assistive services. Today, these technologies are used in diverse healthcare applications and are expected to increase efficacy and efficiency of healthcare providers [6,7]. They target to improve the organization of healthcare providers, improve therapy and rehabilitation, and enhance prevention and care. In the field of dementia assistance, prototypes were developed for specific scenarios to assist patients during different stages of cognitive decline ranging from healthy ageing to severe cognitive impairment [8-10]. Other remote monitoring systems are used for mobility measurement to estimate disturbances in motor activity of the patients to prevent risk of accidents [11]. Some systems use video and audio recording for patient tracking in order to analyze their activities [10,12].

The acceptance of these technologies by dementia patients and their caregivers is a critical factor for the success of such systems. As Bradram et al. [6] reported, *“only few of the proposed context-aware technologies and applications have been deployed in a real use-setting outside the computer science lab for a longer period of time”,* therefore, there are few studies focusing on the acceptance of AAL technologies in the field of healthcare [13,14]. Some results attest that technology acceptance for healthcare is mainly based on the ease of use, usefulness, trust and management support [15,16]. Privacy is also a major factor for the acceptance of technology for healthcare [17]. In current research at the Network Ageing Research group (NAR) in Germany [18], it has been shown that the acceptance of AAL technologies by dementia patients is rather low in the initial stage as maintaining a familiar lifestyle is still possible without help. However, acceptance can increase with progressive symptoms that threaten the independence of the patient.

The dynamism of AAL technologies and their adaptability to heterogeneous environments and different patients’ profiles and needs are among the main technical requirements we identify in this paper. In the literature, one may find work contributing to this field. Existing research on application polymorphism [19] and network selection [20] has helped on the portability of applications through different devices and on the network selection during device mobility. Some work already exists allowing the discovery of, and the interaction with devices [21,22]. The dynamic integration of entities (sensors, devices, services, etc.) based on the use of a middleware and some semantic representation was introduced in a position paper by Helal et al. [23]. However, no environmental bindings are added to the devices in these approaches; thus, the use of devices is only based on their intrinsic characteristics without taking the context of the patient into account. When it comes to sensors, some work has contributed to wide area sensors network self-configuration [24]. However, strong contributions are yet to be found on context binding of newly discovered sensors.

## Rationale of our deployment approach

Our research aims at improving healthcare and quality of life for dementia patients during early stages when cognitive impairments are still mild and amenable to assisted intervention. We are interested in supporting healthcare in nursing homes by helping residents to perform their ADLs and by providing support to caregivers. Therefore, we are focused on developing and deploying a technical solution that will provide assistive services to help residents and their caregivers. This solution is designed and developed from scratch to fulfil the requirements of a clinical environment.

Our approach is to start from a pre-deployment analysis conducted in a nursing home closely with end-users (dementia patients) and specialists in dementia care (professional caregivers) in order to identify the needs, develop and deploy a technical system based on the collected requirements, then evaluate the performance and usability of the proposed solution in real settings. This approach involves healthcare specialists in the design process, as recommended by Orpwood & al [4]. Also, we pushed further the idea to include professionals in the evaluation of the performance and usability of the proposed solutions in real life conditions.

We envision that such a multidisciplinary design approach, supporting a deployment in real life settings is crucial; and that a simple system developed and validated in this way is more relevant and valuable than a well-featured solution proven stable only in a laboratory. In fact, most of the systems in the field of smart homes and dementia assistance work perfectly in a laboratory testing environment; however, they fall short when they progress towards commercialisation or real deployment due to the lack of collaboration with professionals in the domain and the restriction of these studies to laboratories prototyping and testing.

Some ideas for building prototype environments [25,26] are interesting, as they help to involve stakeholders in the design and testing process. However, real life scenarios are unlimited and cannot be enumerated and tested in prototyping environments where there is only a limited number of users and the technology is used only for a short time. In addition,these environments do not help to evaluate the technical usability of the designed system and the reaction of stakeholders in real world settings. Indeed, technical problems (sensors pulled off by patients, bad network connectivity, etc.) and design problems (household routines, multiple users, adaptability of the system to different patients’ profiles, etc.) unpredictable in these environments during the design process and may only appear at the system delivery stage. These problems should be identified and resolved beforehand, during the development phase.

## Methods

### Research approach to real life deployment

#### Choice of deployment environment

We chose to position our study in a nursing home as it simplifies the recruitment of consenting residents and offers a semi-controlled environment, where professional caregivers are present on the field, allowing us to gather feedback and interest of stakeholders. We partnered with a nursing home hosting elderly patients with mild dementia who correspond to our targeted population.

*Peacehaven*, a nursing home from Singapore’s Salvation Army, is our host and partner in the current study. It hosts around 400 patients with dementia ranging from stage 4 to 5 according to the GDS [2]. Residents on the second floor of this nursing home are at dementia stage 5 (moderate) while residents of the third floor have a mild dementia evaluated at stage 4. Each level has eight professional caregivers to assist residents, although residents from the second floor need more assistance and help. We decided to conduct our study on residents with moderate dementia from the second floor where caregivers have a greater need of a solution to reduce their burden. The deployment was planned in three phases preceded by a pre-deployment analysis and test phase as detailed in Table 1.

**Table 1:** Timeline for the development and deployment of our solution in the nursing home (14 months trial)

**Timeline**	**Description**	**Activities**
Mar 2010 - Mar 2011	Observations, discussions & prototyping	Pre-deployment
Apr - June 2011	Prototyping & Demo	
Jul 2011	Application for ethics approval	
Aug 2011	Ethics approval obtained	
Aug - Oct 2011	Initial trial setup and field testing of system	
Oct - Jan 2012	First phase (1 room, 4 months trial)	Deployment
Jan - Feb 2012	Analysis, features update & performance tuning	+
Feb - May 2012	Second phase (1 room, 4 months trial)	Ground truth
May - June 2012	Analysis & questionnaire survey	+
June - Nov 2012	Third phase (3 rooms, 6 months trial)	Data analysis
Nov - Dec 2012	Analysis & questionnaire survey	

#### Pre-deployment observations and discussions

Before starting the deployment, we chose to conduct weekly observation sessions with residents from the nursing home during a three month period and to organise focus group discussions with caregivers. As residents have a common schedule daily, observation sessions consisted of following the daily schedule with two or three of them and participating in weekly group activities (organised with 10 to 15 residents). Table 2 illustrates some of our observations.

**Table 2:** A snapshot of data collected from observation sessions

**Type of observation**	**Observations**
Rooms observation	• Two to three beds in each room
	• One bathroom is attached to each room
	• Food and medications are taken in the common area
	• TV is only available in the common area
ADLs observation	• One resident (level 2) keeps on washing hand or showering for hours
	• Assisting shower is very difficult for nurses
	• Patients forget to turn off the taps
	• Patients need instructions of what to do next
	• Patients need encouragement to initiate activities
	• They forget to continue activities if they were interrupted, e.g. forget to finish eating after going to the toilet
	• They forget things they have already done, e.g., some may shower too often
Group activities observation	• 10 to 15 residents
	• 2 to 4 professional caregivers
	• Participants are divided into small groups
	• Caregivers need to give instructions

These observations were followed by discussions with caregivers in order to gain a comprehensive understanding of the living conditions at the nursing home. Focus group discussions were organised each time with around 5 professional caregivers and doctors to discuss about the collected observations, present some demos and discuss about possible improvements, as well as patients’ and caregivers’ requirements, needs and issues. Discussions were tape-recorded and started with a reporting of main needs based on a live questionnaire with questions collected from the field or previous meetings, a session last for around 2 hours and is dedicated for updates, exchange and interaction, or for the definition and validation of the assistive services to be provided. Collected information were analysed and processed to produce meeting reports. It was difficult to extract meaningful information from discussions with patients due to their dementia. For example, they may speak about events that have happened a long time ago as if they happened during the current day.

#### Participants’ characteristics and selection process

The staff from the nursing home selected potential subjects, in consultation with the doctors, and the appropriate residents were approached to participate in this study. Ill patients with unstable parameters or life-limiting diseases (such as cancer or end stage heart failure) were excluded from the study. Those with pacemakers and other required medical electronic devices for monitoring or treatment (monitors like telemetry, ECG, pulse oximetry, infusion pumps, etc.) have also been excluded. We have selected only patients who could give informed consent in spite of dementia, based on a mental competence assessment of the patient done by a clinician steeped in dementia care, or patients with dementia who had a Legally Appointed Representative (LAR, usually a close relative) who could provide informed consent on their behalf if they lack mental capacity to do so. The staff selected to participate to the study were those who were actively involved in the day to day care of the selected residents, and knew their habits quite well.

Two patients living in the same room and two caregivers were first involved in the study. Later on, 6 other patients from two other rooms (3 in each room) were granted approval to participate to the trial. The eight patients are women living on the second floor of the nursing home and aged between 78 and 92 years. Two of them need minimal assistance (help to walk or to lie on the bed) while the six others need moderate assistance (help to take the shower or to eat). Table 3 gives an overview of the different patients’ profiles.

**Table 3:** Participants profiles

**Patient**	**Age**	**Functional status**	**Room nb**
Patient 1	90	Need minimal assistance^*^	8
Patient 2	92	Need moderate assistance^**^	8
Patient 3	85	Need moderate assistance	8
Patient 4	79	Need minimal assistance	9
Patient 5	87	Need moderate assistance	9
Patient 6	92	Need moderate assistance	11
Patient 7	82	Need moderate assistance	11
Patient 8	78	Need moderate assistance	11

^*^Minimal assistance: Provided assistance consists only on elementary activities (walking, lying, etc.).

^**^Moderate assistance: Provided assistance includes more critical activities (eating, toileting, etc.).

To proceed with the deployment in real settings, the study was ethically approved by the Institutional Review Board (IRB) of the National University of Singapore (NUS) under the number 11–222. After the *Consent Forms* were signed and collected, and before the start of the deployment, more visits were organized to analyse personalised needs of selected patients with recourse to professional caregivers implicated in the study. Several assistive services related to each patient’s problems were identified.

### Deployed system

The deployed system composes of low cost and non-intrusive sensors (e.g. pressure sensors, proximity sensors, vibration sensors, motion sensors), different devices of interaction (e.g. speakers and tablets for the residents, smart-phones and a nursing console for the caregivers), and a centralized compact machine deployed in each room. Sensors are used to monitor the residents and acquire low level context information while interaction devices are used to provide reminders and notifications for patients or caregivers.

**Figure 1:**
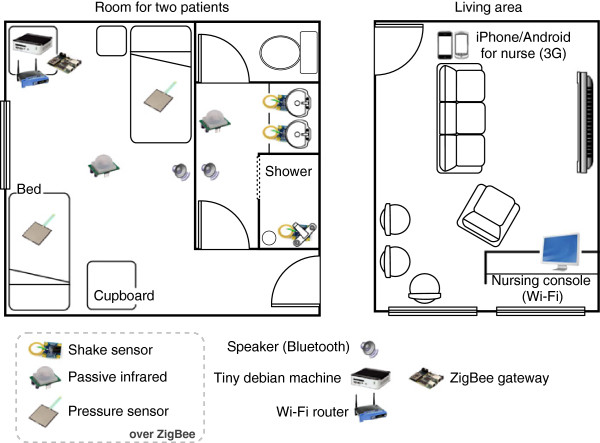
Partial floor map of the nursing home deployment.

The nursing home environment with the different sensors and interaction devices is described in Figure 1. In each room is installed a compact fanless Debian machine (115×115×35 mm, 505 g), mounted with a 500 MB RAM/500 Hz CPU, a 8 GB Compact Flash drive, the whole consuming only 5 W. Sensors are using the ZigBee communication protocol on a wireless sensor network based on Crossbow’s IRIS mote platform. A Crossbow node is connected via serial port to the Debian machine, serving as gateway. The communication with other devices in the environment uses Bluetooth for residents’ embedded speakers, a client-server communication over Wi-Fi for the nursing console (Windows 7 machine with touchscreen) or 3G for the nurses’ smartphones (Samsung Galaxy S2 with Android 2.3 and Apple iPhone 4 with iOS 5).

Within the system, a software platform, assuring the context aware reasoning and the provision of assistive services for the patients, was developed based on human and technical requirements gathered from the pre-deployment analysis, and installed on the Debian compact machine of each room.

### Data gathering & performance evaluation

In order to evaluate the performance of our system, and as we have committed ourselves not to use video recording to preserve the privacy of the nursing home’s residents and caregivers, we have chosen to rely on log-sheets filled by the caregivers and compared with our system logs. We have collected all system log data during our trial period of 14 months and extracted meaningful information concerning sensors states and patients’ context, then compared them with the caregivers’ log-sheets. Log-sheets provide information about the ground-truth: caregivers were asked to fill them up with hourly information about patients’ location, patients’ abnormal behaviours and possible remarks. Table 4 is a sample of blank caregivers’ log-sheet which was used for our ground truth data collection through data entry into a database.

**Table 4:** Sample of caregivers’ log-sheet

**Date: ____________**	**Patient: __________**					
	Where is patient right now?	Did patient shower for too long?	Did patient forget to turn off tap?	Did patient forget to flush toilet?	Did patient wander around aimlessly?	Did patient ask for something?
	-Bedroom	-Yes	-Yes	-Yes	-Yes	-Yes
	-Bathroom	-No	-No	-No	-No	-No
	-Dining area	If so what did (S)he ask for?				
	-Common area					
	-Other					
Hour 1						
Hour 2						
...						

In the *Results* section, we present the outcomes of our pre-deployment analysis and the different technological requirements raised, then we expose our technical contribution consisting in a software platform able to respond to these requirements, e.g. by adapting to different environments and patients’ needs and profiles. Finally we evaluate the performance of the proposed system and we present the results of our data analysis.

## Results

### Outcomes of the pre-deployment analysis

#### Human requirements

From our pre-deployment observations and discussions we gained an understanding of the living conditions at the nursing home and the different problems that residents and caregivers are facing. It was found that although residents were free to move around in the common areas, and in fact did so quite frequently, the majority of the unsupervised time was spent in their bedroom or washroom. Hence the bedroom and its attached washroom were selected as the main areas for our study.

##### Caregivers relief

In our observations, we realised that most of the assistive tasks performed by caregivers consisted of encouraging patients to start some activities, showing them instructions on the first several steps to follow, serving medication or asking patients to drink water. It is usually difficult to follow and assist all the residents’ activities, especially during the night when there are fewer caregivers on duty and patients are prone to more critical situations. Therefore, caregivers considered that reminders and notifications could be helpful for them to be informed on how patients are performing their ADLs, and to provide support as and when appropriate. In order not to increase the attention needed from caregivers, it has been decided that the system should remain silent when no issues are met by patients. Notifications are to be raised only when a reaction is necessary.

##### Independent ageing

Residents were found to have problems like showering repeatedly (some may shower 3 times in the morning because they forget activities they have already done), remain in the shower too long (they stand in the shower and let the water running for an extended period of time because they do not know what to do), wandering (some just walk around in their room during the night and do not go back to sleep) or using other patients’ belongings (sleep on the wrong bed or wear someone else’s clothes). In order to assist the patients in these activities, caregivers emphasised that reminders should first be sent to patients to encourage them to think and retain some level of independence. Caregivers should interfere only when the residents lose their way and are not able to solve their problems independently.

##### Level of dementia

Due to our dedicated design approach and validation environment, and in order to make sure patients experience an optimal engagement and usefulness, a requirement rises on the most suitable level of dementia for assisted patients. Indeed, as introduced earlier, elders who are still able to live purposefully without assistance would not feel the need for such system, hence acceptance would be rather low. On the other hand, the system is designed to help patients keep a certain level of autonomy and therefore rely on their understanding of reminders, e.g. based on their capacity to recognise the purpose of some objects in graphical reminders. This means that patients with a pronounced dementia, in this case an advanced agnosia, would not be able to react to reminders, which would only build up their frustration. From our exchange with clinicians, it has been determined that patients with dementia at GDS level 5 would experience the most suitable assistance.

##### Personalised assistance

Each patient has his own habits and caregivers should assist them in an adapted manner and with different activities. Using our system, caregivers should be able to register an interest that keep track of which services are useful for which residents. Services should also be personalized to the profile and the evolution of the disease of each patient.

Based on these outcomes, we have arrived at possible assistive services that can be provided for the nursing home (using wrong bed, showering too long, showering without soap, wandering, tap left on, fall detection, not sleeping at night), mainly via reminders or notifications. These reminders should be completely based on non-intrusive technologies, especially without any resort to video analysis. We decided to focus on some key assistive services that are interesting for the caregivers and that can be realized by our team to be first demonstrated in the laboratory. Four services (using wrong bed, showering for too long, showering without soap, not sleeping at night) were selected. The nursing home staff appreciated the lab demo and gave positive feedback. The demo was then showcased in real conditions within the nursing home, without including real end-users (caregivers and patients). Feedbacks were mainly focused on the reconfigurability, customisation and adaptability of the system which were starting points for further development of the system.

#### Technological requirements

Discussions and feedbacks from the nursing home staff about the prototypes demonstrated in the laboratory and in the nursing home allowed us to set some technological requirements that a software platform for elders with mild dementia should fulfil.

##### Privacy

Guaranteeing the respect of patients’ and caregivers’ privacy and the confidentiality of the research records is a major requirement. Many systems use video and audio recording to provide assistive services [10,12]; this is however a serious issue when targeting deployment in real settings. Therefore, we are committed to use only non-intrusive sensors, without recourse to any video or audio recording. In addition, collected data are anonymised and personal information are coded and then unlinked from research data whenever any form of processing is done. This means personal information and privacy sensitive data are separated from research data collected throughout the whole deployment. In daily data collection and analysis stages, only sensor data collected with non privacy-sensitive information was used to measure the performance of the system.

##### Multiple people management

Another aspect that needs to be taken into account in a real life deployment is the fact that patients are not living alone. In Singapore, nursing home rooms are usually shared between 2 to 4 residents. Moreover, caregivers need to come into the room to clean and assist the patients. An identification mechanism needs to be established to identify people inside the room and separate their data when possible. We decided to test the use of Radio-Frequency IDentification (RFID) tags for the identification of patients. These passive tags are embedded inside plastic bracelets worn on patients’ wrist. RFID readers are placed at major points of interest such as patients’ beds or on the wash-room entrance. Our system uses information from RFID readers to identify users. When detecting activity in one location without RFID information, the system infers the identity based on partial information from other readers. This process of identification allows us to personalise services for different patients.

##### Design for failure

No matter how much effort is put in the implementation, ambient systems can be prone to crashes. This is partly due to the reliance on several wireless communication protocols. Among others, thick walls, temporary interferences or poor coverage are factors that affect the system’s reliability. We have also met problems related to the use of sensors, being battery issues or simply a curious user pulling off the new “object” in his environment.

When systems are deployed in real settings, there is no more constant supervision by technicians while actual users count on the provided services to maintain their safety. It is becoming very costly to analyse a crash or restart the system as it requires a specifically skilled person to go down to the deployment site. Moreover, if a crash is not detected or crash reports are not sent in a timely manner, this could bring into question the safety of end-users. Hence, the issue of stability takes a whole new dimension in real deployments. Our idea here is that *designing for failure*, which is a nice and luxurious feature for laboratory prototypes, becomes a necessity in real life deployments. *Designing for failure* is based on the idea that crashes are inevitable and should be taken into account during the design and implementation phases. It encompasses the following processes: ensuring the automatic system recovery after crashes, proper logging of information for crashes analysis, and proper signage about the system health for end-users.

##### Dynamism and adaptability of the system

Our experience and more specifically the interaction with stakeholders, has raised an important issue that needs to be tackled in order to deploy a system for assisting dementia patient in their environment. The dynamism and adaptability of the system are crucial to the success of this deployment. Indeed, patients have different behaviours and profiles, with the possibility of progression of their disease. This will have an influence on the choice of interaction devices and assistive services to be provided for each patient. In that sense, and unlike many systems who have only one specific service (or functionality) to provide [8-10], our proposed system is designed to be dynamic and scalable, allowing the integration of new assitive services depending on the needs of each patient.

Integrating new assistive services to the system possibly implies adding new sensors and interaction devices in the environment. The dynamic aspect of the system helps to include them at runtime, after system installation. Targeting a large scale deployment also relies on the dynamic and adaptability aspects of the system. Indeed, it introduces more diversity in patients’ profiles and needs that need to be handled, as well as more heterogeneity of the environments in which sensors, interaction devices and assistive services will be deployed. The system has to be designed so as to improve maintenance efficiency and upgrade speed by simplifying the associated technical processes.

### Specific technical contribution: software platform supporting dynamicity and adaptability

In our approach, we provide an adaptable and dynamic platform, that integrates new assistive services at runtime, and enables their binding to specific patients. It also allows new interaction devices and sensors to be incorporated on the fly. In a similar study supporting dynamic platforms [27], integrated devices and sensors are preconfigured and predefined for specific functionalities; thus, provided services are only based on one sensor and the platform cannot provide assistive services based on multiple sensors. In our approach, assistive services are bound to multiple context information. Hence the platform authorises to attach sensors and interaction devices to several assistive services. It also allows their reconfiguration, when needed, to be used for other assistive services.

Apart from the contributions in more general aspects of our research activity described in this paper, the technical contribution of this work consists mainly on a semantic reasoning mechanism making use of semantic web technologies, and a semantic Plug & Play approach based on the use of the Open Service Gateway initiative (OSGi)^1^ and the Device Profile for Web Services (DPWS) [28] technologies. On one hand, we propose a model allowing the semantic representation of the different entities and the context in a given environment. Based on the evolution of this model as a result of contextual information gathered from sensors, and using first order logic rules, our platform is able to perform some context understanding and select assistive services and interaction devices accordingly. On the other hand, the semantic Plug & Play mechanism allows the discovery of sensors and devices deployed in the environment, their representation into the platform as distinct modules and then their integration into the service selection and provision process at runtime. This allows the system to integrate and provide new assistive services, and adapt to its environment with simple reconfiguration and without recoding or restarting the platform.

#### Semantic context modelling and reasoning

We decided to use semantic web technologies to create our model describing the environment, and especially a semantic reasoner to drive the context-awareness in our platform. This technology allows for a more efficient separation between application logic and underlying models describing the peculiarities of the environment, and brings down to each entity the possibility to understand newly discovered other entities and to create bindings with the environment [29]. These criteria were very crucial in term of re-usability of the platform in personalized environments and its adaptability in dynamic environments. For our implementation, we use Euler [30] as a semantic reasoner. It is notably among the fastest reasoners we have found and is also the most lightweight of the reasoners we selected and compared [29].

**Figure 2:**
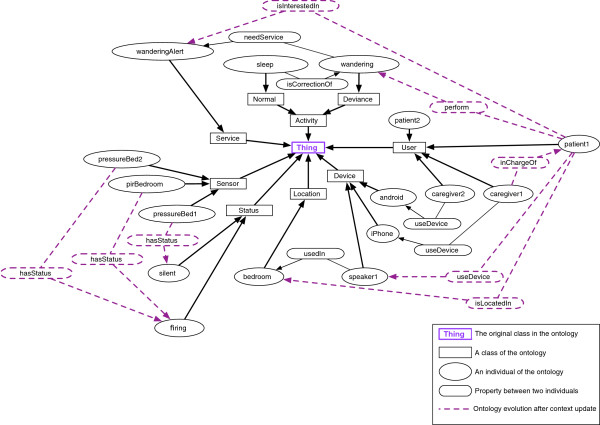
Ontology evolution after a context update.

We provide a semantic model (ontology) that represents the knowledge about entities in the environment (including users and their activities, locations, assistive services, sensors and interaction devices) and the relations between them. Figure 2 shows the evolution of the ontology from an initial state to an updated state resulting from a context change where the patient needs the assistive service wanderingAlert. During the night, patient2 is sleeping while patient1 starts wandering. The relations *(pressureBed 1, hasStatus, silent)* and *(pirBedroom, hasStatus, firing)* are created. The reasoning engine infers that both the two patients are located in the bedroom, and that patient1 is wandering while patient2 is sleeping. The respective relations are created. First order logic rules infer that the patient1 is interested in the wanderingAlert service. As this service is triggered during the night and in order to not disturb other patients, the reminder is not sent on the speaker, and the relation *(caregiver1, inChargeOf, patient1)* is created. A notification is therefore sent to the caregiver1 on his iPhone.

Rules applied by the semantic reasoner are first order logic rules. As an example, the rule below ensures the selection of an appropriate assistive service to provide to the patient, based on the abnormal behaviours we might detect.

$$\begin{array}{lll} & \;\forall \;U\;\text{ser}\;u;\text{Deviance}\;\text{dv};\text{Service}\;s\; & \;\\ & (u,\;\text{per}\;\text{form},\;\text{dv})\;\wedge \;(\text{dv},\;\text{needService},\;s)\; & \;\\ & \;\;\Rightarrow \;(u,\;\text{isInterestedIn},\;s)\; & \; \end{array}$$

#### Semantic plug & play mechanism

In order to ensure the dynamism of the platform, we based our implementation on the use of OSGi, one of the major specifications for the Service Oriented Approach (SOA) which has been used in numerous projects for smart homes development [31-33]. OSGi allows to structure our software platform in a modular way, permitting its flexibility, scalability, reconfigurability, and ease of replication. Using this approach, we represented the different acts of assistance that should be provided for the dementia patient as *assistive services* which can be integrated or removed from the platform at runtime with smooth reconfigurations. This allows caregivers to select assistive services needed for each patient, based on the timely evolution of his cognitive health. The different sensing technologies and interaction devices are also represented into the platform as services (careful: not *assistive services*) that can provide context information or display an assistive service and which can be added, configured or removed on runtime. We chose to use *Apache Felix*^2^, a lightweight implementation of OSGi, which is easy to use in standalone and is suitable for integration in the compact machines we are using for the deployment as it takes up almost 0% of CPU processing time and as low as 12 M of RAM.

Our Semantic Plug & Play approach takes advantage of the DPWS technology and relies on our semantic representation and reasoning to detect new sensors and interaction devices in the environment and integrate them in the reasoning process at runtime. Our implementation allows to build the semantic representation of newly discovered entities once detected, then integrate them into the semantic model. These entities are later bound to specific context (object, location, activity, etc.) through a configuration tool in order to be integrated in the reasoning process. Using the same approach, new assistive services are also added and linked to different sensors and devices [34].

**Figure 3:**
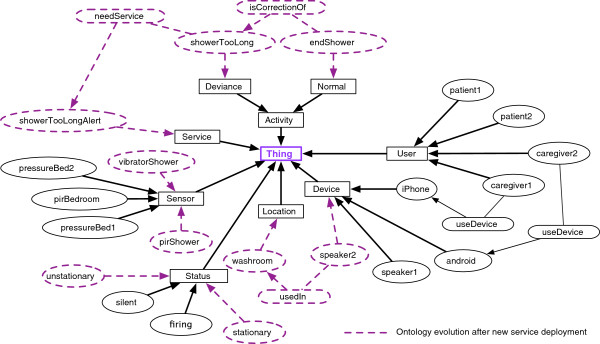
Ontology evolution after new service deployment.

Figure 3, shows an example of a new assistive service (Showertoolong) integration into the platform with its related sensors (pirShower and vibratorShower) and interaction device (speaker2). New relations are also created based on sensors and devices description and configurations from the configuration tool.

### Deployed services

Our solution was deployed in 3 rooms within the pilot site nursing home and includes 8 patients for the trial. The deployment started with an initial trial set-up and field testing of the system for technical validation, without any interaction with the patients and caregivers. Then three phases were planned; during the two first phases the interaction was only with the caregivers to collect feedback and analyse the performance of the system. Patients were not included in the interaction so as to test the system without affecting residents with eventual false alarms. The interaction with patients was initiated during the last phase when we have significantly reduced false alarms generated by the system and increased the system uptime.

We have provided 4 assistive services which were identified with the help of the caregivers from the nursing home based on some issues that participating residents were having the most frequently. We can easily define specific problems for the residents and provide tailor-made assistive services using the dynamic aspect of our software platform. The Four assistive services provided are: 1) wandering at night, 2) showering for too long, 3) leaving the wash-room tap on and 4) toilet fall detection, which not only correspond to actual needs for our residents but also represent dangerous situations when they are in dark or wet environment, and increases the likelihood of a fall. Table 5 gives an overview of these 4 services:

**Table 5:** Assistive services deployed in the nursing home

**Service**	**Sensors**	**Interaction devices**
Wandering at night	Motion sensor	Smart-phones
	Pressure sensor	Nursing
		console
Showering too long	Motion sensor	Speakers
	Vibrator	Smart-phones
		Nursing
		console
Leaving the wash-room	Proximity sensor	Speakers
tap on	Vibrator	Smart-phones
		Nursing
		console
Toilet fall detection	Motion sensor	Smart-phones
	Proximity sensor	Nursing
		console

#### 

##### Wandering at night

Some of our residents usually wake up during the night, go to the toilet, then start wandering in the room without going back to sleep. We have deployed a service to detect this abnormal behaviour. Pressure sensors are deployed under the mattresses of the patients’ beds to detect their presence. During the night, if the patient is not detected on his bed and the motion sensor, placed on the bedroom’s ceiling, is firing, the system infers that the patient is wandering and a notification is sent to the caregivers on their smart-phones and nursing console. There are no interactions with the intended patient to avoid disturbing the co-resident during the night.

##### Toilet fall detection

Falls represent critical situations for the patients and need prompt intervention from the caregivers. They have been found to be more frequent and crucial in the toilet. In order to quickly detect these situations and alert the caregivers, we have deployed motion sensors in the toilet to detect the presence of the patient, as well as attached proximity sensors to the ceiling to measure the height difference of the patient. Once we detect a sudden change in the height, and after a while, if the patient is still in the toilet and the height has not changed, an alert is sent to the caregivers on their smart-phones and nursing console.

##### Showering too long

As explained previously, during the shower, some residents stand in the shower and let the water run for an extended period of time because they do not know what to do. This can be detected by the system, which sends an advice to the patient or a notification to the caregivers. To ensure this, a motion sensor deployed in the shower detects the presence of the patient while the vibrator attached to the water pipeline detects that he has started showering. Another complementary assistive service *shower without soap* was suggested during the pre-deployment period, to detect that patient is not using the soap during the shower, and to notify him to do so. However, caregivers were interested only on the *showering too long* assistive service for the patients involved.

##### Leaving the wash-room tap on

Some patients forget to turn off the tap after washing their hands, which may lead to a flood in the washroom and patients falling down. In order to prevent this, we have deployed a service to detect when the tap is left on. The combination of the proximity sensor to detect the patient near the sink and the vibrator on the water pipeline to detect that the tap is on, infers that the patient is using the tap. Once the vibrator is still firing and the patient is no more near the sink, a reminder is sent to the patient through the speakers to hint to him about turning off the tap. The reminder is repeated for a fixed number of times configurable by the caregivers and personalized for each patient. If the resident ignores the reminders, a notification is sent to the caregivers.

**Figure 4:**
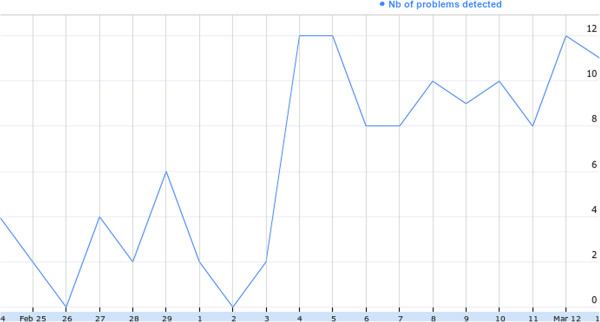
Patient’s detected problems evolution.

Some research work have provided taps that are automatically turned off in case of flooding [4]. This could easily be integrated in our platform but it is not the objective here. Indeed, in our case, patients should be motivated to perform their activities in the right way, and caregivers should be informed about the evolution of their patients disease and how well they are performing their activities. For example, as shown in Figure 4, we have noticed one of our patients situation deterioration. Normally she has less than 6 reminders each day, but suddenly, we have detected an increase of this number up to 12 reminders. This would suggest that the patient’s situation worsened around 4th of March which was confirmed by the caregivers as they have also realised that she had serious problems soon after this period.

The sensitivity of our AAL solution for the detection of these situations correspond to the ground truth data analysis with 71% of confidence during the first phase of the deployment and has reached 83% at the end of the deployment as will be explained later in the System performance section.

In addition to these services, we have developed a *next hour activities prediction service*, based on the data analysis of log sheets from caregivers and system logs. This service allows the prediction of patients activities (bathroom, common area, and room activities) in the next hour using decision tree ensemble and active learning with various parameters. This service was tested with patients 4 and 5 (Room 9) from March to October 2012 (8 months). We have reached an accuracy of 91% and 68% respectively for sleep and toileting activities prediction. This service allows the improvement of caregivers’ planning and time management especially during the night as they can know the patients’ activities an hour in advance and assist when necessary. This gives them more time to care for more patients.

### System performance

Based on the analysis of our system data log, we have classified *atomic* events – e.g. the use of tap, shower and locations – which happen 34 times a day in average, and *complex* events – corresponding to deviances and services provision – which happen 7 times a day in average. The comparison between these results and the ground-truth’s log-sheets during the first phase of the deployment reveals a matching rate of approximately 71% on a weekly basis analysis.

**Figure 5:**
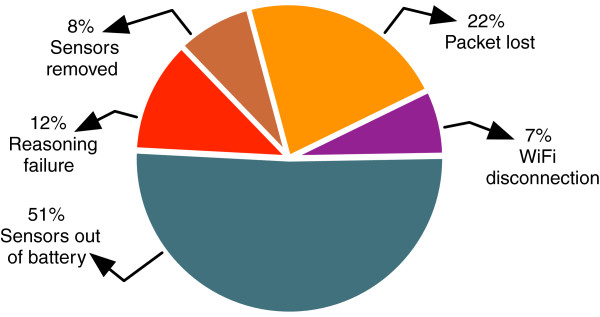
Pie chart for system crash reasons in the nursing home.

The 29% of false alarms detected are related to distinct reasons. They could emerge from human errors caused by caregivers imprecision in the logging time and by the fact that other patients can use the wash-room used for our deployment. At the same time, it is also related to technological failures such as sensors failure (sensor removed, sensor out of battery, sensor packets lost), reasoning failure (software bug) and connection problems (Wi-Fi disconnection, ZigBee communication problem). Our deployment in the nursing home allowed us to highlight these main areas in which the system needs to be improved. Figure 5 shows the relative occurrence of each of these problems from the global number of errors we have detected during this phase. We worked hard on improving these aspects and were able to improve the average uptime of the system from 3 days in December 2011 to 11 days in May 2012. We have also reached an 83% matching rate in the weekly context recognition performance during the last phase of deployment. The more technical errors were considerably reduced, notably the batteries failure, with the use of a new type of batteries and the development of a protocol which involves acknowledge messages to switch off the sensors’ radio usage when it is not used. This has increased the battery life time from 5 days to 15 days. We are also working on improving the reasoning engine performance through formal verification of defined rules using model checking techniques. This allows to detect some unwanted situations such as non-reachable rules which does not affect the system precision but increase the complexity and reasoning time, redundant rules causing multiple decisions about the same situation and logically conflicting rules which can lead to conflicting and non logical decisions [35]. A current work on incorporating a notion of uncertainty in the reasoning process has been initiated to cope with incomplete information derived from faulty hardware, delays between production and consumption of the information, or even networking problems.

Another aspect we have considered as estimation of the performance of our system, is the time needed to set it up into a new environment. Before implementing the dynamic approach and as we had still implemented the context awareness part in an imperative manner, it took 3 days for a team of 2 research-engineers to install and adapt the system in the nursing home environment. Moving to the dynamic approach and the semantic modelling, adaptation to a new environment with its specificities took us only 2 to 3 hours, most of which was to adapt the semantic model. This has been much reduced as the system intrinsic logic is kept unchanged. Once the system is installed, we computed its reaction time; calculated between the time a service is needed and the time it is delivered. This has an average of 2.7s, which has been refined in 1.2s for reasoning process, 0.7s for communication between modules and 0.8s for the processing due to other miscellaneous bundles. The effective time for integrating a new assistive service into the platform was about 2s for the deployment of one assistive service with two sensors and one interaction device.

### Data analysis

From two data sources (caregivers’ log-sheets and system log), we provide two data visualizations of a resident’s behaviour which illustrate that we can provide an early detection of serious problems. This is highlighted through one of our patient’s (Patient 5) data analysis. The nursing home staff informed us that this patient was seriously ill and had to leave the nursing home for medical treatment in late-March 2012, which was detectable in the data gathered and corresponding to the patient’s behaviour in early-March. The proposed visualizations expose the patient’s relative density of activity in bathroom (e.g. pass urine or motion), common area (e.g. watching television or eating), or bedroom (e.g. sleep) when she starts to have medical problems and show the possibility to recognise the deterioration of her situation.

**Figure 6:**
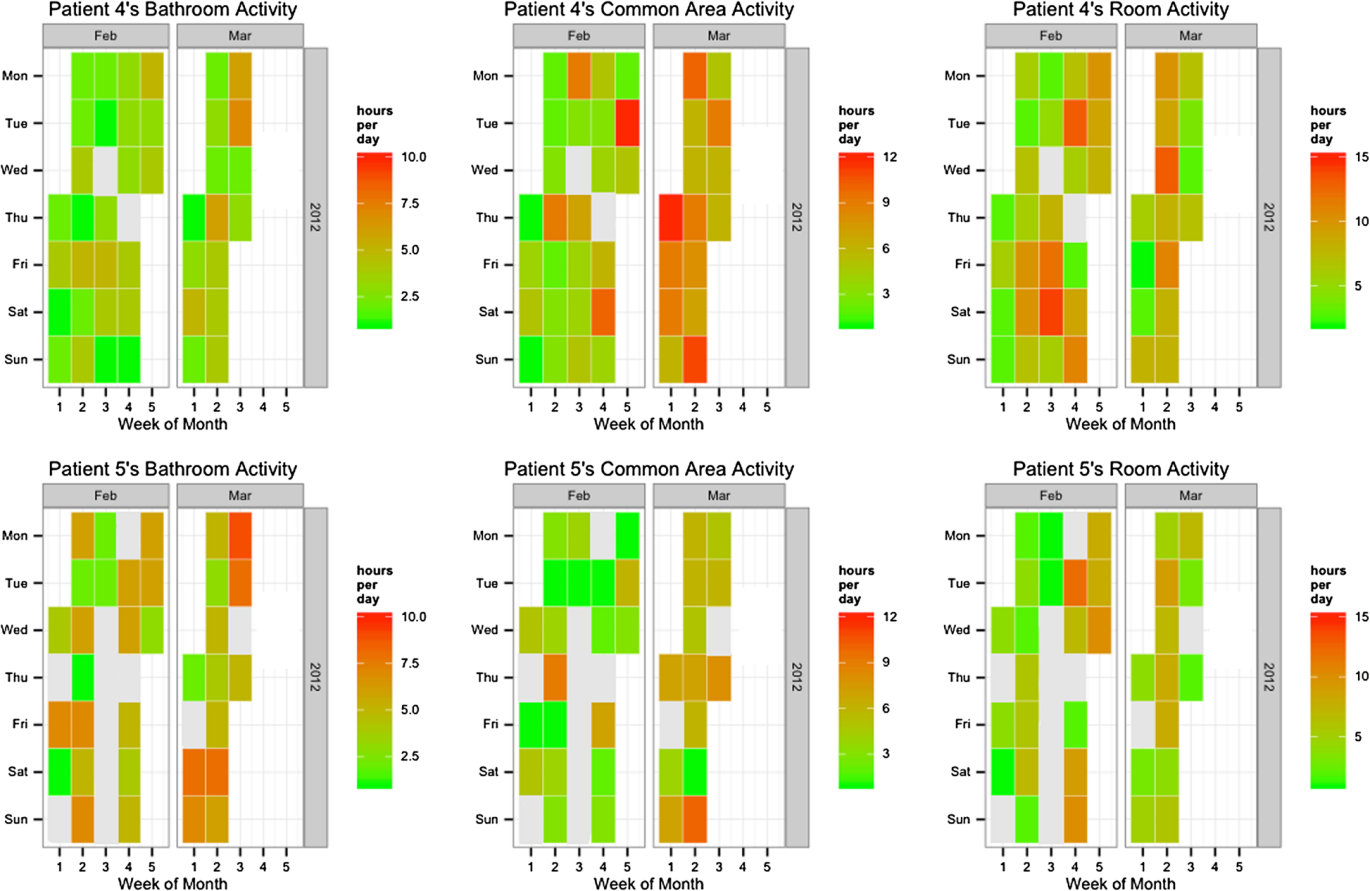
Chronological heatmap of caregivers’ log-sheets (ground truth) for Patients 4 & 5 (Room 9) in February and early-March 2012 (1.5 months).

Figure 6 is a chronological heatmap of caregivers’ log-sheets (ground truth) for patients 4 & 5 in February and early-March 2012 (1.5 months). The comparison of the two patients’ activities demonstrate that patient 5 has significantly more bathroom activities and lesser common area activities (number of orange blocks) and that she spends more days away from nursing home (the gray blocks between colored ones) for hospital treatments. When comparing patient 5’s data across the investigated period, we notice a significant increase in bathroom activities for March 2012 compared to February with more orange blocks. It is very likely that this patient had serious medical problems during this period with more frequent bathroom visits, less ability for leisure activities, and more hospital visits for medical treatment.

**Figure 7:**
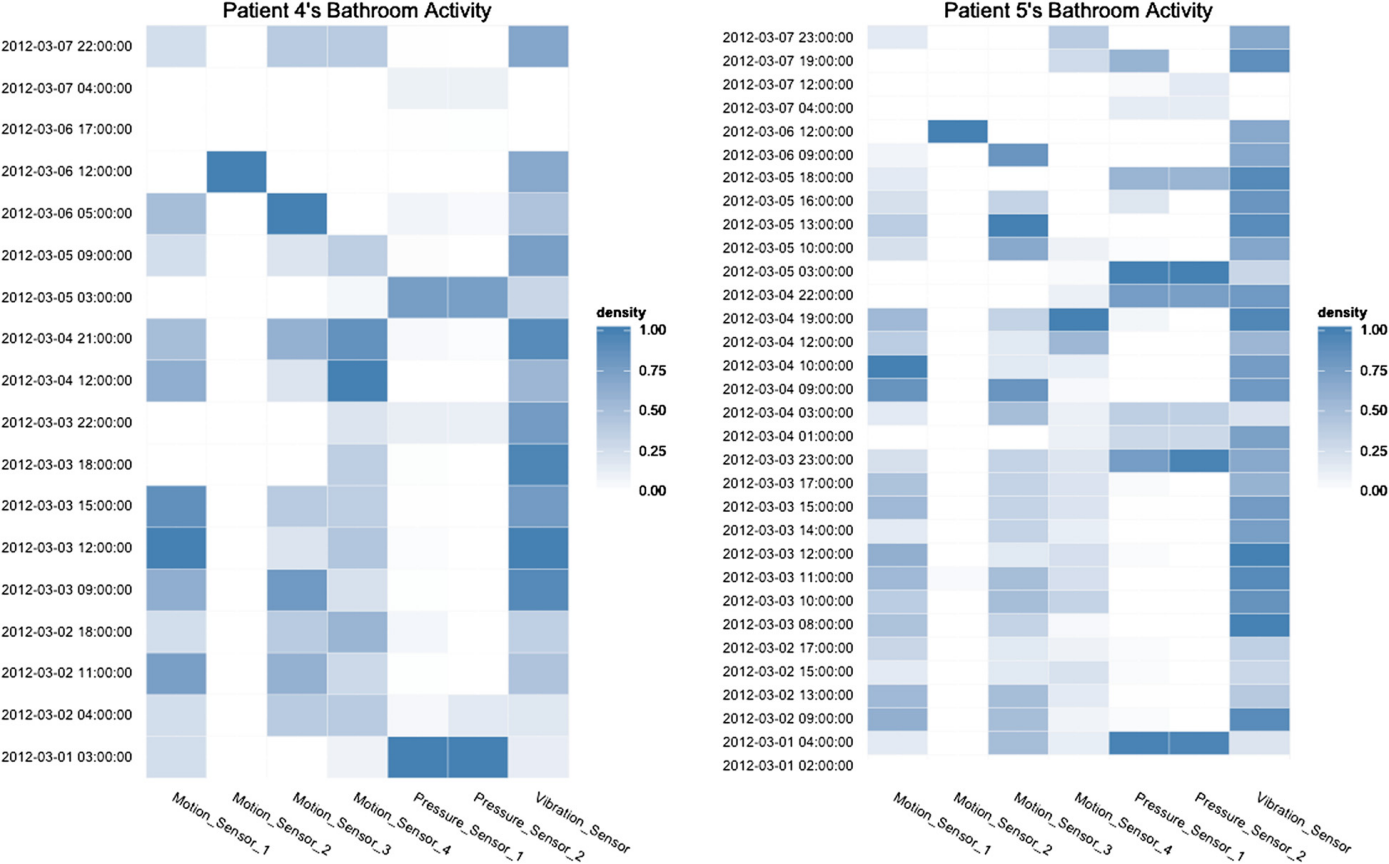
Chronological heatmap of system log data (sensor data) cross-referenced with caregivers’ log-sheets (ground truth) for Patients 4 & 5 (Room 9) in early-March 2012 (7 days) for bathroom activity.

This is also confirmed by Figure 7, which represent a chronological heatmap of system log data (sensor data) cross-referenced with caregivers’ log-sheets (ground truth) for the bathroom activity of the two patients in early-March 2012 (7 days). Visits to toilet are characterized by high amounts of vibration and motion sensor firings while sleep is related to pressure sensor firings. When comparing Patient 4 with 5 for 7 days, Patient 5 has almost twice as many bathroom activities as a normal resident.

### Qualitative feedback

The feedback received from the Nursing Home staff has been encouraging. The deployment was promising in terms of demonstrated features and capabilities. In view of the expected benefits, the head of the Nursing Home remarked that the staff would like to have the full system deployed in every room. This was encouraging since there was a perceptible change in attitude over the months during which the deployment had taken place. The staff caregivers had become more adept in the use of smart phones and appreciated the value of the underlying sensor based technology. Though the staff complied with the completion of the manual log sheets, which was crucial as it was the only form of ground truth available in the absence of video camera based logs, they admitted having difficulties to cope systematically with the extra work it represented. We discussed simplifying the logging process by providing a more automated logging media through tablets embedded in the environment and bringing logging down to a few clicks on touch-screens. Doctors were positive about the deployment and felt that it would go a long way to improve resident safety and add to the well-being and comfort of the residents (P Yap, personal communication, Jan 15, 2013).

## Discussion

In this paper, we have presented the approach we have adopted to develop and deploy an ambient assistive living system for patients with dementia. This approach was based on a pre-deployment analysis with implication of professional caregivers to set different human and technical requirements that the system should fulfil.

From a human perspective, requirements consist mainly of providing adapted assistance with different activities based on each patient’s habits and profile. This assistance encourages the patient to start/stop some activities or show instructions on the first several steps of each activity. Caregivers need this kind of assistance mostly in bedrooms where patients spend the majority of their days and during the night when there are less caregivers. Patients, whose dementia should be at GDS level 5 for an optimal experience, need to perform their activities in the right way and caregivers should be informed how well they are doing. Therefore, the provided assistance should incite patients to think and retain some level of independence in performing their activities without just solving the problems. Caregivers should interfere only when the patient cannot resolve his problem.

From a technical perspective, the system should guarantee the privacy of patients and caregivers, and manage multiple people in controlled areas. It should also be reconfigurable, customisable and adaptable allowing caregivers to select different assistive services for selected residents and adapt them to patients’ profiles and diseases’ evolution. Caregivers consider that reminders and notifications are helpful to provide assistance to patients and inform them about their situations. In order to cope with technical problems resulting from faulty hardware or network problems, the platform should be designed for failure that will allow system recovery, crash analysis and crash reports.

The real life deployment approach adopted not only allowed us to fix these different requirements but also proved to be a source of different lessons learnt. In fact, different challenges have arisen from our deployment in real settings. Problems such as batteries life, sensors removal or network connection cannot be identified while performing experiences in the laboratories under optimum conditions. Deployment allowed us to expose the reasoning engine to more realistic and complex scenarios, thus leading to more robust and concrete performance evaluation. In addition, the involvement of professional caregivers in the design and evaluation process was very helpful, in making sure we provide a system that could respond to stakeholders’ and end-users’ needs and helps with the awareness and acceptability of such systems.

Based on the ability of the platform to detect specific behaviours – and using implicit information contained in sensor data as well –, we hope to provide services that would help to detect long term shifts in the habits of patients as this represent a meaningful clinical element to provide to doctors and/or family members. It has been shown in the Data analysis section that some hindsight are already available as we can predict a few days in advance the degradation of a patient’s condition. At first, we considered gathering manual log-sheets filled by nurses on the field to get some approximated ground-truth that would help us evaluate the system’s recognition performance. However, as these log-sheets were as parse as our sensor data, it was very difficult crossing the data to estimate the performance. The interesting point though, is that we unexpectedly realised that data from manual nurses’ log sheets was at least as valuable as our sensor data, in the sense that by using the same kind of algorithms we were able to extract very meaningful information about patients’ habits and health. One of our major lessons learned is that providing a more automated, electronic and pervasive manner to gather this data from nurses on the field would be a very useful feature to integrate into further AAL solution; naturally with backend algorithms analysing this data and making valuable clinical knowledge available.

The adopted deployment approach requires a lot of time and manpower which may cause deliverables delays. In fact, we were working on solving technical problems and adopting new approaches that are required for real life deployments in order to increase the system accuracy form 71% to 83% and uptime from 3 to 11 days. In addition, the use of some technologies such as semantic web and OSGi is beneficial to guarantee the dynamism and adaptability of the system but requires specific skills with a time consuming learning curve. In order to increase the system uptime, sensor batteries lifetime was increased from 5 to 15 days, which is still considered to be short during the deployment. We are trying out a different sensor platform and up until now, we have reached 4 months of autonomy.

Despite our deployment of interactive devices for the residents and the extension of the trial from two to eight patients, the number of participants is still limited, hence a significant amount of data is missing to provide meaningful results. Further surveys concerning the impact on social and health aspects are needed, and should be realised during future larger scale deployments as we now have a system that is quite stable, and quickly deployable and adaptable to different environments. Still a significant contribution is provided in this paper, as we demonstrated the system’s efficiency to detect signs of health deterioration and abnormal behaviours of residents. The qualitative feedback and lessons learned provided can also serve as inspiration for developers of assistive living services.

## Conclusion

In this paper, we have presented an approach to deploy assistive technologies, that starts from a pre-deployment analysis on the field in a collaborating nursing home with implication of professional caregivers in order to gather human and technical requirements. We have then provided a high-level overview of a software platform that responds to these requirements which was deployed in our collaborating nursing home for 14 months with 8 patients and 2 caregivers. An analysis of the data collected during this deployment allowed us to demonstrate the ability of the proposed solution to detect abnormal behaviours and to evaluate the performance of the system with the help of caregivers. The deployment also helped us to extract a set of lessons learned that will serve for further research work in the domain.

## Endnotes

^1^http://www.osgi.org/.

^2^http://felix.apache.org/.

## Competing interests

The authors declare that they have no competing interest.

## Authors’ contributions

HA is the main contributor to this paper which represent his PhD research work supervised by MM. MM conceptualised and supervised the research work. TT contributed to the overall work and specifically to the reasoning part. HA and TT designed and implemented the platform. JB supervised the sensors networking activities which was implemented by LJHK. CF contributed on the data analysis. PY provided his expertise in the medical support and contributed to the experimentation in PeaceHaven nursing home. All authors read and approved the final manuscript.

## Pre-publication history

The pre-publication history for this paper can be accessed here:

http://www.biomedcentral.com/1472-6947/13/42/prepub
